# CD8 T-Cell Induction against Vascular Endothelial Growth Factor Receptor 2 by *Salmonella* for Vaccination Purposes against a Murine Melanoma

**DOI:** 10.1371/journal.pone.0034214

**Published:** 2012-04-12

**Authors:** Stefan Jellbauer, Klaus Panthel, Justin H. Hetrodt, Holger Rüssmann

**Affiliations:** 1 Max von Pettenkofer-Institute for Hygiene and Medical Microbiology, Ludwig-Maximilians-University Munich, Munich, Germany; 2 Department of Microbiology and Molecular Genetics, University of California Irvine, Irvine, California, United States of America; 3 Institute for Microbiology, Immunology and Laboratory Medicine, HELIOS Clinic Emil von Behring, Walterhöferstrasse, Berlin, Germany; Cincinnati Children's Hospital Medical Center, United States of America

## Abstract

The *Salmonella* type III secretion system (T3SS) efficiently translocates heterologous proteins into the cytosol of eukaryotic cells. This leads to an antigen-specific CD8 T-cell induction in mice orally immunized with recombinant *Salmonella*. Recently, we have used *Salmonella*'s T3SS as a prophylactic and therapeutic intervention against a murine fibrosarcoma. In this study, we constructed a recombinant *Salmonella* strain translocating the immunogenic H-2D^b^-specific CD8 T-cell epitope VILTNPISM (KDR2) from the murine vascular endothelial growth factor receptor 2 (VEGFR2). VEGFR2 is a member of the tyrosine protein kinase family and is upregulated on proliferating endothelial cells of the tumor vasculature. After single orogastric vaccination, we detected significant numbers of KDR2-tetramer-positive CD8 T cells in the spleens of immunized mice. The efficacy of these cytotoxic T cells was evaluated in a prophylactic setting to protect mice from challenges with B16F10 melanoma cells in a flank tumor model, and to reduce dissemination of spontaneous pulmonary melanoma metastases. Vaccinated mice revealed a reduction of angiogenesis by 62% in the solid tumor and consequently a significant decrease of tumor growth as compared to non-immunized mice. Moreover, in the lung metastasis model, immunization with recombinant *Salmonella* resulted in a reduction of the metastatic melanoma burden by approximately 60%.

## Introduction

Attenuated recombinant *Salmonella enterica* serovar Typhimurium has emerged as a promising delivery system for foreign vaccine antigens [1], [2]. Upon close contact with the eukaryotic cell, a type III secretion system (T3SS) encoded by the “*Salmonella* pathogenicity island 1” mediates *Salmonella* invasion of the host cell, where the bacterium resides within *Salmonella*-containing vacuoles. The T3SS is designed to translocate *Salmonella* type III effector proteins directly into the cytosol of target cells [3]. Our laboratory has focused its research on the genetic manipulation of attenuated *Salmonella* strains to endow them with the ability for efficient induction of MHC class I-restricted immune responses [4]. We have developed a new vaccination strategy by using the *Salmonella*-T3SS to translocate microbial antigens directly into the cytosol of antigen-presenting cells. The immunodominant p60 antigen from *Listeria monocytogenes* was fused to the defined N-terminal translocation domain of the type III effector molecule *Yersinia* outer protein E (YopE) [5]. Translocation and cytosolic delivery of the chimeric YopE/p60 protein into macrophages led to efficient MHC class I-restricted antigen presentation of the p60 nonamer peptide p60_217–225_. As determined by enzyme-linked immunospot assay, mice orally vaccinated with a single dose of attenuated *Salmonella* expressing translocated YopE/p60 protein revealed high numbers of interferon-gamma (IFN-γ)-producing CD8 T cells reactive with p60_217–225_. In more recent studies, we demonstrated that the use of *Salmonella*'s T3SS to induce antigen-specific cytotoxic T cells is also an attractive strategy to develop vaccines for the immunoprophylaxis of tumors [6] and is also suitable as a therapeutic intervention against cancer [7]. For these experiments, we have established an experimental tumor model in BALB/c mice [6], [7]. The murine fibrosarcoma cell line WEHI 164 [8] was stably transfected with DNA encoding the immunodominant listerial MHC class I-restricted nonamer epitope p60_217–225_ [6]. In naïve mice, subcutaneous inoculation of these WEHI-p60 tumor cells resulted in progressive growth of a solid fibrosarcoma for approximately 14 days without inducing a measurable frequency of p60_217–225_-tetramer-positive CD8 T cells [6]. BALB/c mice received a single orogastric immunization with *Salmonella* that translocates YopE/p60 via its T3SS [6]. Four weeks later, mice were challenged subcutaneously with WEHI-p60 tumor cells. *In vivo* protection studies revealed that 80% of these mice remained tumor-free, whereas all animals of the non-vaccinated control group developed tumor growth. Furthermore, the potential of this vaccination strategy was evaluated with regard to a therapeutic intervention against cancer [7]. Therefore, we used the above described model and applied the YopE/p60 expressing *Salmonella* vaccine strain either simultaneously or 4 days after subcutaneous tumor injection. Interestingly, 71–80% of the intravenously and 50–52% of the orogastrically immunized mice showed a complete tumor regression after 14 days. In addition, the distribution of tetramer-positive p60_217–225_-specific CD8 T cell subpopulations in blood and tumor tissue was analyzed [7]. Co-staining with CD62L and CD127 revealed that the frequencies of p60_217–225_-specific effector and effector memory CD8 T cells in blood and in fibrosarcoma tissue were related to the kinetics of tumor regression.

Tumors have evolved multiple mechanisms to evade the immune response, including antigen loss, down regulation of the MHC, and the production of immuno-suppressive factors [9], [10]. In addition, many tumors lack expression of co-stimulatory molecules critical for the activation of naïve T cells. Finally, tolerance mechanisms are operative *in vivo* to prevent T cell activation in response to tumor antigens that are expressed in many cases also in normal tissue. A new approach to circumvent some of these problems, is the idea to suppress tumor angiogenesis by using various types of anti-angiogenic agents [11]. Angiogenesis is a critical mechanism for tumor progression [12]. The vascular endothelial growth factor (VEGF) family of proteins is one of the most potent and specific positive regulators of angiogenesis. These proteins bind to three tyrosine kinase receptors, vascular endothelial growth factor receptor 1 (VEGFR1/Flt-1), VEGFR2 (KDR/fetal liver kinase 1, Flk-1), and VEGFR3 (Flt-4) [13]. VEGFR2 is the major mediator of mitogenic, angiogenic, and permeability-enhancing effects of VEGF in endothelial cells [14]. VEGFR2-null mice die in utero between days 8.5 and 9.5 with no signs of vasculogenesis or organized blood vessels [15]. Thus, VEGFR2 plays a crucial role in the processes of vascularization and angiogenesis. Recent studies in mice have shown that tumor angiogenesis can also be inhibited when cellular immune responses are induced against vascular endothelial growth factor receptor 2 (VEGFR2) [16], [17].

In this study, we present a novel vaccination strategy to induce VEGFR2-specific CD8 T cells in mice orally vaccinated with attenuated recombinant *Salmonella enterica* serovar Typhimurium. Induction of cytotoxic T cells targeting VEGFR2-positive endothelial cells of the tumor vasculature was achieved by cytosolic delivery of the H-2D^b^-specific CD8 T-cell epitope VILTNPISM ( = KDR2) from the murine VEGFR2 using *Salmonella*'s T3SS [18]. The efficacy of the *Salmonella* T3SS-based vaccination against VEGFR2 was evaluated in a prophylactic setting in mice that received B16F10 melanoma cells in their right flank. In additional experiments, the same immunization strategy was used to reduce dissemination of melanoma pulmonary metastases. In both tumor models, we could show that vaccination against VEGFR2 reduced angiogenesis and tumor growth significantly, highlighting the potential of this novel vaccination protocol for cancer immunotherapy.

## Materials and Methods

### Ethics Statement

Animal experiments were approved by the German authorities (Regierung von Oberbayern, Bavaria, Germany; approval IDs 55.2-1-54-2531.8-08-06 and 55.2-1-54-2531-125-05) and performed according to the legal requirements.

### Plasmids, bacterial strains, and growth conditions

The construction of plasmid pHR241 has been outlined in detail [5]. This low-copy-number expression vector bears the genetic information for the translocated chimeric YopE_1–138_/p60_130–477_/M45 fusion protein under expression control of the *lac* promoter which is constitutively active in *Salmonella*. M45 is derived from the E4-6/7 protein of adenovirus and its use for chimeric protein tagging has been described [5]. Plasmid pHR584 is a derivative of pHR241 and encodes for a hybrid YopE_1–138_/VEGFR2_352–411_ protein. This plasmid was generated by using standard PCR cloning procedures. Template DNA for VEGFR2/Flk-1 was kindly provided by Andreas Niethammer (University of Heidelberg, Germany) [17]. Plasmid pHR584 was transformed into *Salmonella enterica* serovar Typhimurium strain SB824 by electroporation. Strain SB824 was engineered by introducing the *sptP::kan* mutant allele from strain SB237 into the Δ*aroA* strain SL3261 by P22HT*int* transduction [4], [19], [20]. Serovar Typhimurium was grown in Luria-Bertani medium supplemented with 0.3 M NaCl, pH 7.4, to allow expression of components and targets of the T3SS encoded by the *Salmonella* pathogenicity island 1 which mediates *Salmonella* invasion of host cells [21].

### Detection of secreted hybrid YopE protein by Western Blot analysis

Secreted proteins from *Salmonella* culture supernatants were prepared as follows. Briefly, bacterial supernatants were passed through a 0.45 µm pore-size syringe filter to remove bacteria. Protein in the bacteria-free medium was precipitated by the addition of cold trichloroacetic acid to 10% (v/v) and incubated for 2 h on ice. The protein was collected by centrifugation at 4°C, 10,000× *g* for 20 min. Pellets were washed in 0.8 ml cold acetone, dried, and resuspended in phosphate-buffered saline (PBS) buffered with 80 mM Tris-HCl, pH 8. Samples corresponding to 500 µl culture supernatant were separated in a 10% discontinuous sodium dodecyl sulphate polyacrylamide gel electrophoresis (SDS PAGE), and transferred to nitrocellulose membranes. Hybrid proteins were detected by immunoblot analysis. Western blots were treated with a polyclonal antibody against YopE followed by incubation with a horseradish peroxidase-labeled anti-mouse antibody. Blots were developed by using a chemiluminescence kit.

### Orogastric immunization of mice with recombinant *Salmonella*

Specific-pathogen-free female C57Bl/6 mice, 6–8 weeks old, were purchased from Harlan-Winkelmann (Horst, Netherlands). For the experiments, animals were housed in groups of five mice under standard barrier conditions in individually ventilated cages (Tecniplast, Buguggiate, Italy). Mice were pre-treated orally with 20 mg Streptomycin 24 h prior to *Salmonella* infections to reduce the bacterial gut flora and to enhance uptake of the vaccine strain [22]. Water and food were withdrawn for 4 h before groups of mice were orally immunized with 5×10^8^ colony-forming units (CFU) of the indicated *Salmonella* vaccine strain by using round bottom gavage needles. Thereafter, drinking water ad libidum was offered immediately and food 2 h post immunization. Animal experiments were approved by the German authorities and performed according to the legal requirements.

### Analysis of *Salmonella* loads in small intestine, Peyer's patches, mesenteric lymph nodes, livers and spleens

At the indicated time points post immunization, mice were euthanized by CO_2_ asphyxiation, and samples from the small intestine (SI), Peyer's patches (PP), mesenteric lymph nodes (MLN), livers and spleens were removed for analysis. Organs were weighed before resuspending them in 500 µl of 4°C phosphate-buffered saline, pH 7.4. Then, organs were homogenized in 4°C phosphate-buffered saline, pH 7.4, either manually or using metal beads in an automatic homogenizer (Retsch, Wuppertal, Germany). The numbers of CFU per 1 g were determined by plating appropriate dilutions on Luria-Bertani agar plates containing ampicillin at 100 µg/ml. It is noteworthy that pHR584 was remarkably stable *in vivo*, with 98% of the bacterial population retaining the plasmid 14 days after infection (data not shown).

### Generation and purification of H2-K^d^ tetramers

The generation of tetrameric H2-K^d^/KDR2_400–408_ complexes has been outlined in detail [23]. Briefly, recombinant H2-K^d^ heavy chain and β_2_-microglobulin were expressed as insoluble inclusion bodies in *Escherichia coli* and were further purified. The H2-K^d^ heavy chain molecule was mutated to remove the transmembrane and cytosolic domain and to add a specific biotinylation site at the C-terminus. Purified proteins were refolded *in vitro* in the presence of high concentrations of synthetic peptides (Biosythan, Berlin, Germany) to form stable and soluble MHC/peptide complexes. Complexes were specifically biotinylated *in vitro* by adding the enzyme BirA, d-biotin, and ATP. After further purification, biotinylated MHC/peptide complexes were multimerized with streptavidin-PE (SA-PE; Molecular Probes, Eugene, OR). Tetrameric complexes were purified by gel filtration and stored at 2–5 mg/ml at 4°C in phosphate-buffered saline (pH 8.0) containing 0.02% sodium azide, 1 µg/ml pepstatin, 1 µg/ml leupeptin, and 0.5 ml EDTA.

### Preparation of cells from spleens

At the indicated time points, spleens were removed from animals, harvested by dissociation through a wire mesh, and subsequently resuspended in RP10+, which consists of RPMI 1640 (Invitrogen, Karlsruhe, Germany) supplemented with 10% fetal calf serum, l-glutamine, HEPES (pH 7.5), 2-mercaptoethanol, penicillin (100 U/ml), streptomycin (100 µg/ml), and gentamicin (50 µg/ml).

### MHC tetramer staining and FACS analysis

The KDR2_400–408_-specific T cell populations were detected with phycoerythrin (PE)-conjugated, tetrameric MHC I/peptide complexes and concurrently stained for other surface molecules using directly conjugated monoclonal antibodies as described previously [23], [24]. Briefly, cells were incubated with ethidium bromide monoazide for live/dead discrimination in FACS-staining buffer (phosphate-buffered saline pH 7.45, 0.5% bovine serum albumin, and 0.02% sodium azide). Subsequently, cells were stained with the above mentioned MHC class I tetramer and surface markers for 1 h. The following monoclonal antibodies were used: anti-CD8α (clone 53-6.7, BD, Heidelberg, Germany), anti-CD62L (clone Mel-14, NatuTec, Frankfurt, Germany), and anti-CD127 (clone A7R34, NatuTec). Cells were resuspended in staining buffer and fixed in 1% paraformaldehyde/phosphate-buffered saline (pH 7.45). Data were acquired on a FACSCanto II (BD Biosciences, San Jose, CA) and further analyzed with FlowJo software (TreeStar, Ashland, OR). C57Bl/6 mice were orogastrically immunized with a single dose of 5×10^8^ SB824 (pHR584). Data are displayed as relative numbers of H2-K^d^/KDR2_400–408_-tetramer-positive CD8 T cells that were normalized on the background tetramer staining in spleens of non-immunized mice.

### Flank tumor model

Subconfluent B16F10 cells [25] that were purchased from ATCC (Manassas, VA, USA) were trypsinized, washed, and resuspended in RPMI-1640 (Invitrogen, Karlsruhe, Germany). The cell suspension (3×10^5^ cells in 100 µl) was injected subcutaneously into the right flank of C57BL/6 mice. Groups of five mice were used and randomly divided. Tumor growth was monitored by measuring the length and width of the individual tumors using a calliper [6], [7]. At the end of the experiment, mice were euthanized by CO_2_ asphyxiation and tumors were removed for immunohistological analysis.

### Artificial metastasis model

Subconfluent B16F10 cells [25] were trypsinized, washed, and resuspended in RPMI-1640. The cell suspension (2×10^5^ cells in 100 µl) was injected intravenously into the tail vein of C57BL/6 mice in order to mimic the metastatic process. Mice were observed for symptoms of illness and two weeks after the injection euthanized by CO_2_ asphyxiation and lungs were removed and analyzed for metastases. Using Photoshop software, the number of metastases and the metastases-covered lung area were calculated.

### Histology and immunohistochemistry

Melanoma tumors were prepared for cryosection and 7 to 10 µm cryosections were stained with a FITC-conjugated CD31 (PECAM-1, Becton Dickinson, Heidelberg, Germany) antibody and DAPI. Additionally, HE staining was performed on cryosections. Stained cryosections were analyzed using the Olympus BX61 immunofluorescence microscope. The phase analysis and particle detection program of Cell∧P software from Olympus were used for measuring the fluorescence intensity and the area of the CD31-FITC staining, respectively.

### Statistical analysis

The statistical significance of the results was checked with the non-parametric Mann-Whitney-U-Test at the 0.005 significance level, or as indicated. All tests were performed using the SigmaStat software (Systat Software GmbH, Erkrath, Germany).

## Results

### Construction of chimeric YopE_1–138_/VEGFR2_352–411_ and analysis of its secretion efficacy by Western Blotting

**Figure 1:**
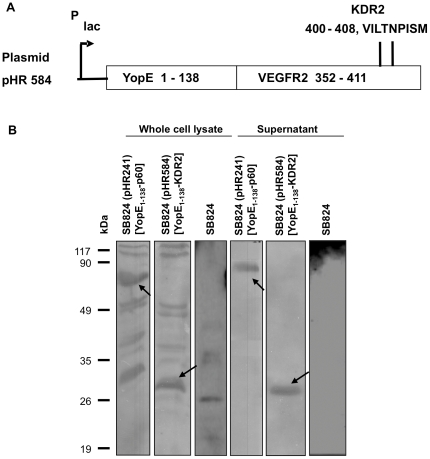
Construction of chimeric protein YopE_1–138_/VEGFR2_352–411_ and its secretion efficacy. **A** Schematic representation of the translational fusion of YopE_1–138_ and VEGFR2_352–411_ encoded by plasmid pHR584. **B** Western blot analysis of hybrid protein YopE_1–138_/VEGFR2_352–411_ showing its stable production and efficient secretion into the *Salmonella* culture supernatant. SB824 (pHR241) expressing YopE_1–138_-p60 served as a positive and plasmid-less SB824 as a negative control.

To obtain a *Salmonella* vaccine strain for anti-angiogenic immunotherapy, a protein fragment containing the immunodominant KDR2 (VILTNPISM) epitope of the murine VEGFR2 was fused to YopE. YopE is a type III effector protein of *Yersinia enterocolitica* and acts as a carrier molecule to translocate the translational fusion protein into the cytosol of antigen-presenting cells (e.g. macrophages and dendritic cells) [5]. The N-terminal 138 amino acids of YopE containing the secretion and translocation domains [26], [27] were fused to amino acids 352 to 411 of Flk-1 [17]. Thus, the newly constructed low-copy-number plasmid pHR584 bears the genetic information for chimeric YopE_1–138_/VEGFR2_352–411_ including the nonamer epitope KDR2 (VEGFR2_400–408_) (Fig. 1a). The bacterial whole cell lysate in Fig. 1b reveals that the constitutive expression of the respective gene fusion by the *lac* promotor led to the stable production of the hybrid protein by *Salmonella*. Furthermore it is shown that chimeric YopE_1–138_/VEGFR2_352–411_ is efficiently secreted into the *Salmonella* cultural supernatant. Previously, we have demonstrated that secretion of YopE_1–138_/p60 correlates with T3SS-dependent translocation of this hybrid protein into the cytosol of macrophages by serovar Typhimurium [5]. As demonstrated in Fig. 1b, both YopE_1–138_/p60_130–477_ and YopE_1–138_/VEGFR2_352–411_ are produced and secreted in comparable amounts by *Salmonella*. As expected, in the supernatant of plasmid-less SB824 (negative control) no protein fragment was detected.

### Time course of colonization and persistence of *Salmonella* SB824 (pHR584) in mouse tissues

**Figure 2:**
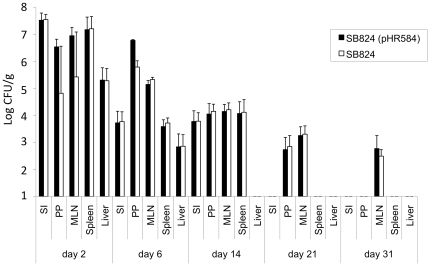
Colonization kinetics in different organs after oral vaccination with *Salmonella* SB824 (pHR584) or SB824. Small intestines (SI), Peyer's Patches (PP), mesenteric lymph nodes (MLN), spleens and livers of five mice were analyzed for the amount of *Salmonella* (determined as colony-forming units per gram tissue; CFU/g) on days 2, 6, 14, 21 and 31 post immunization. Each experiment was performed at least twice with similar results.

In a recently published study, we orally immunized BALB/C mice with the attenuated serovar Typhimurium *aroA*/*sptP* mutant strain SB824 (pHR241) expressing translocated YopE_1–138_/p60_130–477_, and the kinetics of colonization and persistence of the bacteria *in vivo* were investigated [28]. In the current study, C57Bl/6 mice were orogastrically vaccinated with 5×10^8^ SB824 (pHR584) or SB824 and the time course of colonization was determined by counting the numbers of viable bacteria, as CFU per g tissue, in the small intestine (SI), in Peyer's Patches (PP), in mesenteric lymph nodes (MLN), in the liver and the spleen on days 2, 6, 14, 21, and 31 after immunization. No significant differences between the colonization kinetics of both *Salmonella* strains were detected. As shown in Fig. 2, SB824 (pHR584) and SB824 were already recovered from all mouse tissues two days after orogastric application. From day 2 to day 6 the amount of *Salmonella* in all organs decreased significantly. On day 14, both strains could not be isolated from the liver anymore, whereas in all other tissues salmonellae were still detected. On days 21 and 31, SB824 (pHR584) and SB824 were still present in MLN albeit in very few numbers. Taken together, the newly constructed vaccine strain rapidly colonized intestinal mouse tissues after orogastric immunization and persisted for at least 14 days.

### KDR2-specific CD8 T-cell response after single orogastric immunizations

**Figure 3:**
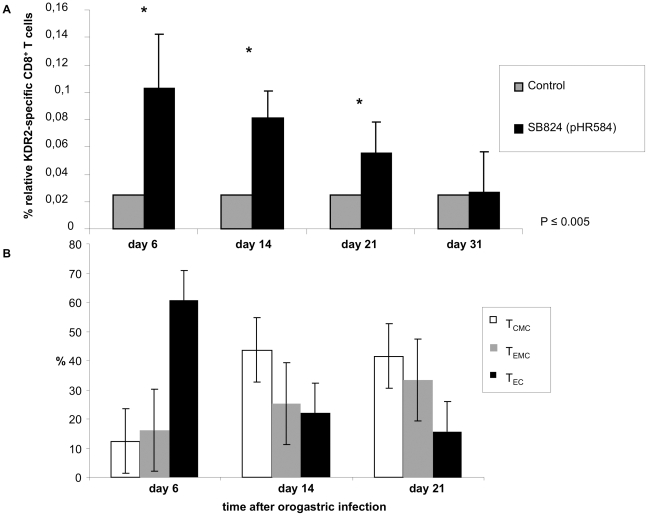
KDR2-specific CD8 T-cell response and kinetics of KDR2_400–408_-specific CD8 T cell subpopulations. **A** MHC tetramer staining using tetrameric H2-Kd/KDR2_400–408_ complexes and flow cytometric analysis of murine spleen cells were performed on days 6, 14, 21 and 31 after single orogastric immunization with *Salmonella* SB824 (pHR584). Relative numbers of H2-K^d^/KDR2_400–408_-tetramer-positive CD8 T cells are shown for five mice vaccinated with SB824 (pHR584) (black bars) and for five non-immunized mice (grey bars, control). Asterisks indicate values that differ significantly from the corresponding values obtained from mice of the control group (*, *p*≤0.005). **B** Differentiation patterns of KDR2_400–408_-specific CD8 T cells into phenotypically distinct subsets on days 6, 14, and 21 after single orogastric vaccination with *Salmonella* SB824 (pHR584) are displayed as % of KDR2_400–408_-specific CD8 T cells. KDR2_400–408_-specific CD8 T cells were stained for expression of CD127 and CD62L and analyzed by flow cytometry. CD127^high^/CD62L^high^ central memory CD8 T cells, T_CMC_, white bars; CD127^high^/CD62L^low^ effector memory CD8 T cells, T_EMC_, grey bars; CD127^low^/CD62L^low^ effector CD8 T cells, T_EC_, black bars. Each experiment was performed at least twice with similar results.

The potential of attenuated serovar Typhimurium expressing chimeric YopE_1–138_/VEGFR2_352–411_ to induce KDR2-specific CD8 T cells *in vivo* was investigated. For this purpose, C57Bl/6 mice were orogastrically immunized with a single dose of 5×10^8^ SB824 (pHR584). Control mice remained non-immunized. After 6, 14, 21, and 31 days, spleens of vaccinated mice were removed and the relative numbers of H2-K^d^/KDR2_400–408_-tetramer-positive CD8 T cells were assessed. In Fig. 3a (white bars) it is demonstrated that the highest level of KDR2-specific CD8 T cells (0.102%±0.01) was detected as early as 6 days after immunization followed by a gradual decline of antigen-specific T cell frequencies on days 14 and 21. On day 31, no significant numbers of KDR2-specific CD8 T cells were detected like in non-vaccinated mice (black bars). In summary, orogastric immunization with SB824 (pHR584) can be used to induce a rapid KDR2-specific CD8 T cell response within 6 days after prime vaccination.

### Co-staining of KDR2_400–408_-specific CD8 T cells with CD62L and CD127 to determine differentiation patterns into phenotypically distinct CD8 T cell subsets

Tetrameric H2-K^d^/KDR2_400–408_ complexes were used to characterize the development of KDR2-specific effector (T_EC_), effector memory (T_EMC_) and central memory (T_CMC_) CD8 T cells in spleens of immunized mice by co-staining with CD62L and CD127. T_CMC_ reside preferentially in lymphoid organs and lack immediate effector functions [29], whereas T_EMC_ migrate mainly into non-lymphoid tissue and elicit immediate effector functions on antigen reencounter. Recent results indicate that interleukin-7 receptor α-chain (CD127) surface expression is a marker for long-living memory T cells [30]. The combination of surface staining for CD127 and L-selectin (CD62L) further separates between T_CMC_ (CD127^high^/CD62L^high^) and T_EMC_ (CD127^high^/CD62L^low^) allowing to distinguishing T_EC_ (CD127^low^/CD62L^low^) from memory T cells at early time points of *in vivo* immune responses.

In our study, C57Bl/6 mice were orogastrically immunized with a single dose of 5×10^8^ SB824 (pHR584). Spleens from vaccinated mice revealed T_EC_ as the predominant KDR2-specific CD8 T-cell subset (60±9.99%) 6 days after vaccination, whereas memory T cells (T_EMC_ and T_CMC_) represented only 15±3.72% and 12±12% of these specific CD8 T cells, respectively (Fig. 3b). However, analysis of the KDR2-specific CD8 T cell subpopulations on day 14 showed 45±16.85% T_CMC_ and 25±13.27% T_EMC_. Thus, the subset of T_EC_ was significantly reduced to 22±18.67%. Three weeks after orogastric immunization, >70% of KDR2-specific CD8 T cells were CD127-positive and thus revealed a memory T cell phenotype (T_CMC_ 40±14.86% and T_EMC_ 32%±18.12%, respectively). Taken together, the analysis of the kinetics of KDR2-specific CD8 T cell subpopulation formation revealed a clear trend towards the induction of memory T cell subsets.

### Vaccination with *Salmonella* SB824 (pHR584) leads to reduced angiogenesis in the melanoma flank tumor model

**Figure 4:**
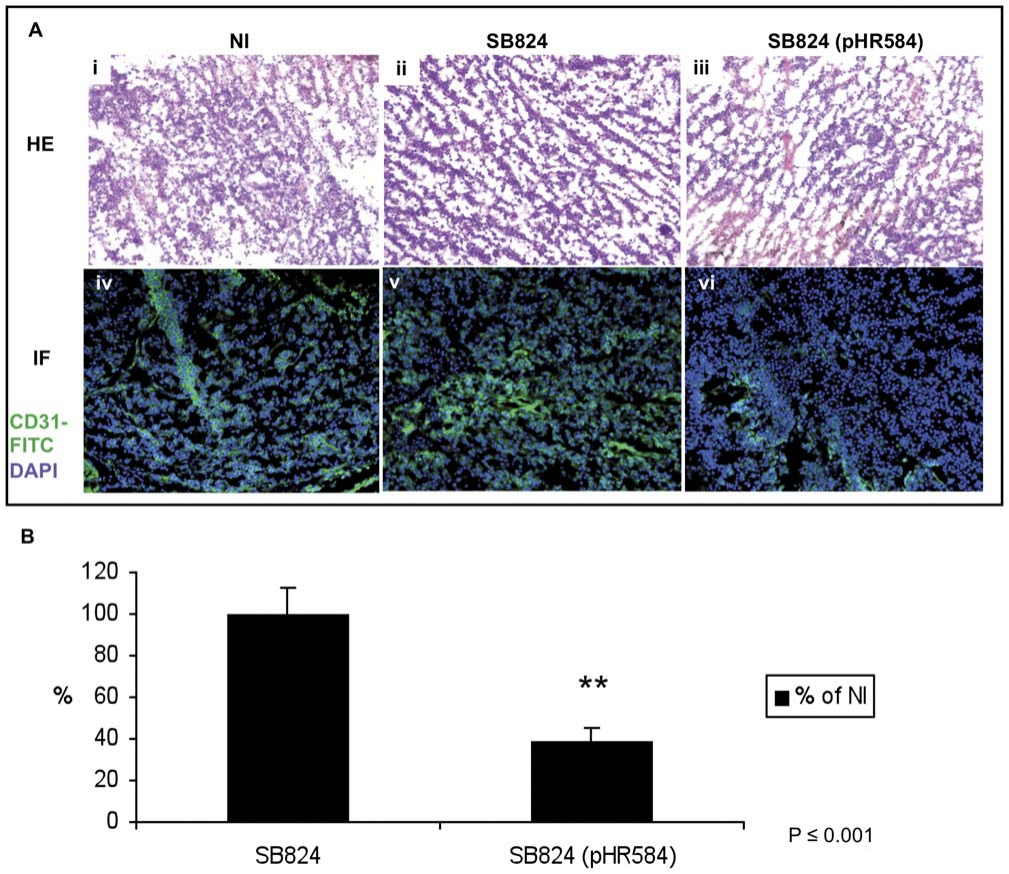
Vaccination with *Salmonella* SB824 (pHR584) leads to reduced angiogenesis in the melanoma flank tumor model. **A** On day 44 of the experiment, melanoma tumors of five mice were removed, prepared for histological analysis and stained with HE (i–iii). Additionally, tumor sections were stained with a FITC-conjugated CD31 antibody (green) and DAPI (blue), and analyzed by immunofluorescence microscopy (iv–vi). Representative images of tumor sections from three experimental groups are displayed. **B** Quantification of the area of the green FITC-fluorescence was performed using Cell∧P software (Olympus). The mean areas of CD31-FITC fluorescence from tumor sections of experimental groups SB824 and SB824 (pHR584) are shown as fraction of the mean area of CD31-FITC fluorescence from tumor sections of the non-immunized control group in %. Asterisks indicate values that differ significantly from the corresponding values obtained from mice of both control groups (**, *p*≤0.001). Each experiment was performed at least twice with similar results.

Previously, Niethammer *et al.* have shown that DNA vaccination against VEGFR2 resulted in a CD8 T cell-dependent reduction of tumor angiogenesis and tumor growth [17]. In a next set of experiments, we addressed the question whether vaccination of C57Bl/6 mice with the attenuated *Salmonella* strain SB824 (pHR584) and the subsequent induction of KDR2_400–408_-specific CD8 T cells reduces the formation of new blood vessels in the melanoma flank tumor model. As above, C57Bl/6 mice were orogastrically immunized with a single dose of 5×10^8^ SB824 (pHR584). Control mice received plasmid-less SB824 or remained non-immunized. On day 30, 3×10^5^ B16F10 melanoma cells were applied subcutaneously into the flank of mice and tumor growth was monitored for two weeks. At the end of the experiment, on day 44, tumors from mice of all three groups were prepared for histological analysis. Haematoxylin and eosin (HE) staining revealed the typical structure of melanoma tumor tissue (Fig. 4a, i–iii). Endothelial cells are an essential part of blood vessels and express CD31 (platelet/endothelial cell adhesion molecule, PECAM-1) [31]. Thus, CD31 staining reveals the amount of blood vessels in tumor sections. We performed immunofluorescence microscopy of tumor cryosections stained with a FITC-conjugated CD31 antibody and DAPI to visualize cell nuclei (Fig. 4a, iv–vi). At a first glance, tumor sections of mice vaccinated with SB824 (pHR584) revealed reduced green fluorescent areas (Fig. 4a, vi) in comparison to tumor sections of control mice (Fig. 4a, iv and v) suggesting a reduced angiogenesis in the melanoma tissue. Images shown in Fig. 4a are representative for approximately 50 tumor cryosections from different mice of each experimental group. The impression of decreased angiogenesis in tumors of mice immunized with SB824 (pHR584) could be verified by measuring the fluorescence intensity and the area of the CD31-FITC staining with the phase analysis and particle detection program of Cell∧P software from Olympus. The mean area of the CD31-FITC fluorescence in tumors of non-immunized mice was defined as 100%. Tumors of mice immunized with plasmid-less SB824 revealed exactly the same fluorescence area (Fig. 4b). However, this area was strikingly decreased to 38% in tumors of mice that were vaccinated with SB824 (pHR584).

### Vaccination with SB824 (pHR584) leads to reduced melanoma growth in the flank tumor model

**Figure 5:**
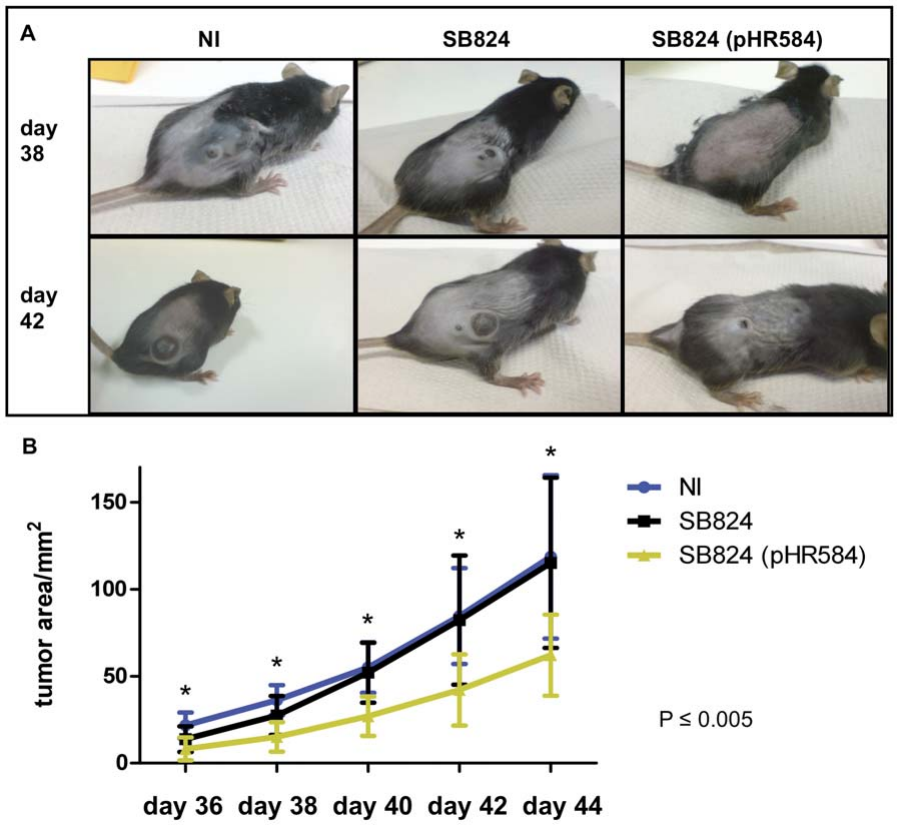
Significant reduction of melanoma tumor growth by *Salmonella*-mediated vaccination against VEGFR2. **A** Photographs of the clinical outcome of C57Bl/6 mice with subcutaneous B16 melanoma tumors. From left to right representative mice from experimental groups NI, SB824 and SB824 (pHR584) on day 38 and day 42 are shown. **B** Tumor growth was monitored over time and the mean tumor area was calculated. Asterisks indicate values that differ significantly from the corresponding values obtained from mice of both control group (*, *p*≤0.005).

To investigate the effect of *Salmonella*-mediated tumor vaccination against VEGFR2, we monitored melanoma growth in the above described subcutaneous tumor model. Again, C57Bl/6 mice were orogastrically immunized with a single dose of 5×10^8^ SB824 (pHR584). Control mice received plasmid-less SB824 or remained non-immunized. On day 30, 3×10^5^ B16F10 melanoma cells were applied subcutaneously into the flank of mice and tumor growth was determined on days 36, 38, 40, 42, and 44. Representative pictures of the clinical outcome of tumor development on days 38 and 42 are shown in Fig. 5a. Obviously, vaccination with SB824 (pHR584) significantly reduced melanoma tumor growth in C57Bl/6 mice. In fact, at every time point of tumor measurement, the mean melanoma areas in mice immunized with SB824 (pHR584) were significantly smaller (approximately 50%) than in mice of both control groups (Fig. 5b). For example, on day 44, the tumor area in mice vaccinated with SB824 (pHR584) was 62 mm^2^ (±23 mm^2^), whereas in both control groups the melanoma areas were 120 mm^2^ (±47 mm^2^). Interestingly, 1 out of 10 mice immunized with SB824 (pHR584) remained tumor-free until day 44, while all 20 mice of the both control groups developed tumor growth. Taken together, these data suggest that vaccination of mice with SB824 (pHR584) significantly reduces growth of subcutaneously applied melanoma tumor cells.

### Vaccination with *Salmonella* SB824 (pHR584) reduces dissemination of melanoma pulmonary metastases

**Figure 6:**
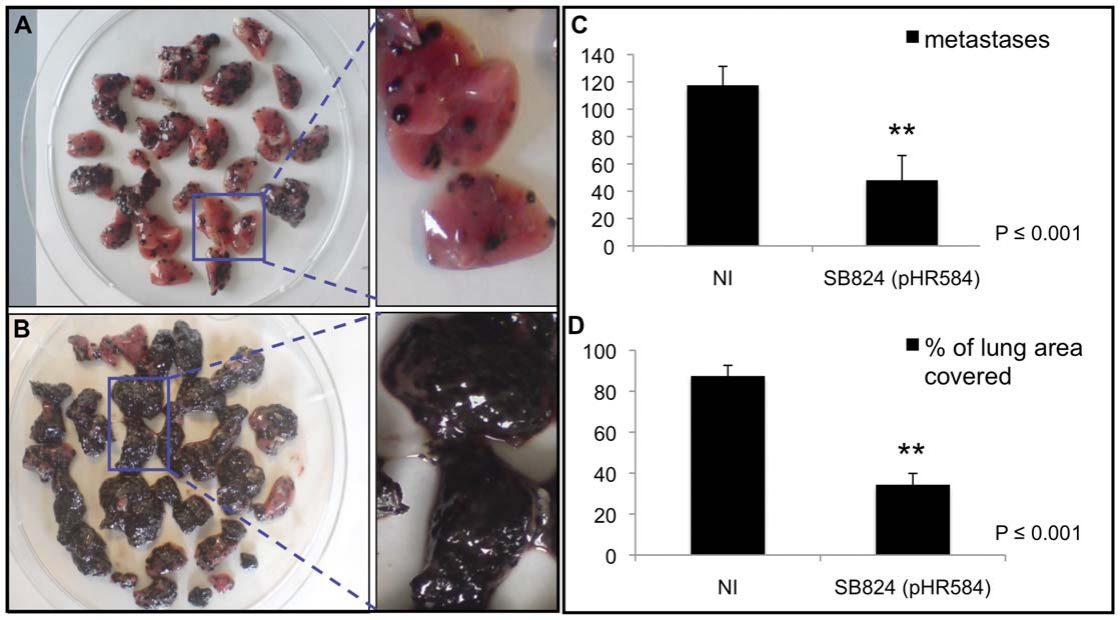
Analysis of lung metastasis after *Salmonella*-mediated vaccination against VEGFR2. **A** In the artificial lung metastasis model, C57Bl/6 mice were orogastrically vaccinated with SB824 (pHR584) followed by a tumor challenge with an intravenous injection of B16 melanoma cells on day 30 in order to mimic the metastatic process. Lungs of ten mice vaccinated with SB824 (pHR584) and magnification of single lobes are displayed. **B** Lungs of ten non-immunized mice and magnification of single lobes. **C** Average number of metastatic spots on lungs (**, *p*≤0.001) **D** Average percentage of surface area of the lungs covered with metastases (**, *p*≤0.001). Each experiment was performed at least twice with similar results.

Tumor vaccination using *Salmonella* SB824 (pHR584) was also tested in a B16 melanoma metastasis model. Therefore, C57Bl/6 mice were orogastrically vaccinated with SB824 (pHR584) followed by a tumor challenge with an intravenous injection of B16 melanoma cells on day 30. Mice of the control group remained non-immunized. Two weeks after the challenge (day 44), lungs were prepared from sacrificed mice and analyzed for metastatic growth. Fig. 6a and b show macroscopic views of the lungs and magnified single lobes from ten mice of both experimental groups. Remarkable differences in the appearance of the lungs were obvious. Lungs of non-immunized mice seemed to be almost totally covered with melanoma metastases, whereas lungs of vaccinated mice revealed only single metastatic spots on their surfaces. Using Photoshop software, the amount of metastases and the surface area of the lungs covered with metastases could be determined (Fig. 6c and d). In lungs of mice immunized with SB824 (pHR584), the average metastatic burden (48 spots) was reduced by approximately 60% as compared to non-immunized mice (118 spots) (Fig. 6c). About 35% of the lung surface areas from mice of the former group were covered with metastases, whereas the lungs from mice of the latter group revealed almost 90% coverage with metastases (Fig. 6d). Interestingly, the health condition of mice vaccinated with SB824 (pHR584) appeared to be unaltered. In contrast, non-immunized mice showed signs of sickness like anorexia and malnutrition during the time of metastatic dissemination. Mice vaccinated with plasmid-less SB824 revealed a comparable metastatic burden and similar signs of sickness as non-immunized mice (data not shown). Thus, a *Salmonella*-mediated unspecific “bystander-effect” responsible for reduced dissemination of pulmonary metastases is unlikely.

## Discussion

Live attenuated bacteria (e. g. *Salmonella enterica*, *Listeria monocytogenes* and *Mycobacteria*) have been widely used as vaccine carriers for foreign antigen display to induce cell-mediated immunity [1], [2]. In the past, the T3SS of *Salmonella enterica* serovar Typhimurium became an attractive tool for heterologous protein delivery directly into the cytosol of macrophages and dendritic cells resulting in vigorous antigen-specific CD8 T cell priming and the induction of protective immunity against viruses, bacteria and tumors [4]–[7]. Unquestionable, CD8 T cells play a pivotal role in the host defence against viruses, intracellular bacteria and tumors. However, the induction of potent CD8 T cell responses to fight microbes or cancer remains a major challenge in vaccine development. Here we show that T3SS-mediated antigen delivery by *Salmonella* can be used for vaccination against VEGFR2 to block tumor angiogenesis.

Tumor angiogenesis is a very attractive target for cancer therapy for several obvious reasons. In general, angiogenesis is required for tumor growth implicating that an anti-angiogenic vaccination approach can be theoretically used to fight a number of different tumor types [16], [17]. Targeting endothelial cells instead of cancer cells benefits from the genetic stability of benign cells in contrast to malign tumor cells. Endothelial cells are not associated with mutations or downregulation of MHC molecules for antigen presentation [16]. Furthermore, endothelial cells of the tumor vasculature are readily and better accessible for therapeutics (effector T cells, antibodies) via the bloodstream than tumor cells [32]. Eventually, targeting endothelial cells for tumor therapy bears a multiplicity effect because each tumor vessel supplies hundreds of tumor cells [33].

VEGFR2, which is upregulated on proliferating endothelial cells of the tumor vasculature, plays an essential role in tumor neoangiogenesis and is therefore an ideal target for anti-angiogenic therapy approaches [16], [17]. The importance of VEGFR2 in antitumor therapy was demonstrated in several studies using different strategies. Besides approved drugs like the monoclonal antibody bevacizumab targeting VEGF-A [34], diverse studies in preclinical and clinical trials efficiently showed the antitumor effect of fixed xenogeneic endothelial cells [35], VEGFR2-conjugated proteins [36], VEGFR2-pulsed dendritic cells [16], [32], liposomal peptides of the fibroblast growth factor-2 [37], several DNA-vaccines against survivin, integrin β_3_, VEGFR and VEGFR2 [33] and viral vectors for DNA-vaccines [38]. Niethammer *et al.* could show that DNA-vaccination can block tumor angiogenesis by induction of a T cellular immune response against VEGFR2 [17]. This DNA-vaccine efficiently protected mice from the growth of melanoma, colon carcinoma and small lung cell carcinoma cells and reduced metastasis.

Our novel vaccination strategy to induce VEGFR2-specific CD8 T cells in mice is based on a recombinant *Salmonella* strain that delivers the H-2D^b^-specific CD8 T-cell epitope VILTNPISM ( = KDR2) from the murine VEGFR2 directly into the cytosol of antigen-presenting cells via the bacterium's T3SS. In mice orally immunized with the *Salmonella* vaccine strain SB824 (pHR584), KDR2-specific CD8 T cells aimed to target endothelial cells were directly monitored by KDR2-MHC class I-tetramer staining. The latter technique was further used to characterize the development of KDR2-specific effector, effector memory and central memory CD8 T cells in murine spleens by co-staining with CD62L and CD127 [30], [39]. Effector and memory T cells display diversity with respect to their effector functions, homing potential, and proliferative capacity [40]. T_EC_ dominate the expansion phase, migrate to peripheral organs, and display immediate effector function. T_EMC_ preferentially home to peripheral tissues and respond to antigen encounter with immediate effector function but poor numeric expansion [41]. In contrast, T_CMC_ home to lymphoid organs, can vigorously expand upon antigen reencounter and are therefore potentially assigned to the crucial T cell population that confers long-lasting protective immunity against microbial pathogens [42]. However, proliferative capacity does not necessarily correlate with protection against infection, since T_CMC_-like CD8 T cells induced by vaccination with heat-killed *L. monocytogenes* reveal vigorous proliferation and expansion after challenge with live *Listeria*, but do not protect against listeriosis as determined by clearance of the bacteria [43]. These findings were supported by a recent study demonstrating that T_EMC_ are more effective than T_CMC_ in conferring protection in the murine *Listeria* infection model [41], [44]. As mentioned above, the analysis of the kinetics of KDR2-specific CD8 T cell subpopulation formation after SB824 (pHR584) vaccination revealed a clear trend towards the induction of both memory T cell subsets. Proliferation of tumor cells appears less fast as bacterial cell division. It is tempting to speculate that KDR2-specific T_CMC_ and T_EMC_ constitute an ideal pair of CD8 T cell subsets to tackle cancer cells. However, further experiments such as functional *ex vivo* assays analyzing the killing activity and cytokine production are needed to characterize KDR2-specific CD8 T cells and their impact on the observed anti-tumor effects.

In further experiments, the efficacy of *Salmonella* T3SS-based vaccination was evaluated in a prophylactic setting to protect mice from lethal challenges with B16F10 melanoma cells in a xenograft flank tumor model, and to reduce dissemination of spontaneous pulmonary metastases in a B16 melanoma model. As compared to non-immunized mice, immunofluorescence staining of flank tumor sections with an antibody against CD31 revealed a reduction of angiogenesis in the solid melanoma by 62% in mice vaccinated with SB824 (pHR584). Most likely, this effect was mediated by KDR2-specific CD8 T cells that specifically recognized and destroyed VEGFR2-positive endothelial cells of the tumor vasculature. As a consequence, tumor growth was significantly reduced in vaccinated mice by approximately 50%. Niethammer and colleagues have previously demonstrated a comparable anti-angiogenic effect using a DNA-vaccination strategy against VEGFR2 [17]. They observed a reduction of angiogenesis by 70% in a colon carcinoma model. Dong *et al.* used a vaccination approach with KDR peptides and reported a reduction of angiogenesis by 70%, too [18]. Similarly to our *Salmonella*-based vaccination strategy, this peptide-mediated immunization decreased tumor growth by 50%.

In the artificial lung metastasis model, immunization with SB824 (pHR584) resulted in a reduction of the metastatic melanoma burden by approximately 60%. An even higher rate of reduction (∼85%) was demonstrated by Li and colleagues using dendritic cells pulsed with VEGFR2-peptid for vaccination purposes [16]. In our experimental set-up, about 35% of the lung surface areas from mice vaccinated with SB824 (pHR584) were covered with metastases, whereas the lungs from non-immunized mice showed 90% coverage with metastases. DNA vaccination against VEGFR2 by Niethammer *et al.* also counteracted the process of metastasis. Less than 20% of the surface area of murine lungs was covered with metastases as compared to more than 50% in non-vaccinated mice [17]. Thus, interference of specific CD8 T cells with angiogenesis impedes the efficient formation of metastases.

Since VEGFR2 is also expressed at lower levels in normal vascular endothelium, vaccination against this receptor could theoretically cause side effects or an autoimmune response. In the DNA vaccination approach of Niethammer *et al.* the wound healing was delayed following vaccination but the fertility of mice was not affected [17]. Controversially, Li *et al.* did not observe a delay in wound healing by dendritic cell (DC)-based vaccination against VEGFR2. However, the authors found an inhibitory effect of DC-VEGFR2 immunization on fetal development [16]. Finally, Dong *et al.* did not detect any obvious side effects following vaccination with antigenic peptides against VEGFR2 [18]. In our study, we did not detect any obvious side effects either.

An interesting question arises from the observation that the vaccine strain SB824 (pHR584) was recovered from the gut, lymph nodes and even the spleen as early as two days after orogastric application. What is the site of KDR2-specific CD8 T-cell priming? Further experiments are needed to determine whether these antigen-specific T cells are derived from the gut, and then disseminate to the spleen or to the site of tumor growth, or, alternatively, are stimulated in lymph nodes.

Interestingly, Nishikawa *et al.* observed the phenomenon of epitope spreading using the *Salmonella* T3SS as an antigen delivery system [45]. The authors engineered a serovar Typhimurium strain expressing the human germ cell antigen NY-ESO-1 via the T3SS. NY-ESO-1 is often expressed by tumor cells but not by normal somatic cells. Oral administration of serovar Typhimurium NY-ESO-1 to mice led to the regression of established NY-ESO-1-expressing tumors. However, intratumoral inoculation of this *Salmonella* strain into NY-ESO-1-negative tumors resulted in the delivery of the antigen *in vivo* and led to tumor regression in the presence of preexisting NY-ESO-1-specific CD8 T cells. In addition, specific T-cell responses against at least two unrelated tumor antigens not contained in the vaccine strain were observed, demonstrating epitope spreading. In future experiments we will investigate whether the phenomenon of epitope spreading also influences *Salmonella* T3SS-mediated vaccination against VEGFR2.

This is the first report demonstrating that *Salmonella* T3SS-mediated antigen delivery can be used for efficient VEGFR2-specific CD8 T cell induction. This novel method of cancer immunotherapy might complement or even boost other anti-angiogenesis immunization strategies such as DNA or peptide vaccination. To confirm the general potential of our *Salmonella*-based vaccination strategy to elicit an anti-tumor effect, we plan to use several tumor lines in future experiments. Interestingly, is has recently been demonstrated for the first time that a VEGFR2-expressing subset of T regulatory T cells (Tregs) exists [46]. It is conceivable that VEGFR2 might become a new therapeutic target for controlling Tregs with highly suppressive function in tumor immunotherapy. Furthermore, two recently published studies have identified blood vessels grown directly from cancer cells suggesting that tumor cells build-up their own vasculature using a mechanism related to cancer-stem cells [47], [48]. These new findings reinforce the need for alternative anti-angiogenic vaccination strategies.
